# Lung function monitoring in patients with duchenne muscular dystrophy on steroid therapy

**DOI:** 10.1186/1756-0500-5-435

**Published:** 2012-08-13

**Authors:** Darlene L Machado, Elaine C Silva, Maria B D Resende, Celso R F Carvalho, Edmar Zanoteli, Umbertina C Reed

**Affiliations:** 1 Department of Neurology, Medical School of the University of São Paulo, Av. Dr. Enéas de Carvalho Aguiar 255, room 5131, Cerqueira Cesar, São Paulo, 05403900, Brazil; 2 Department of Physical Therapy, Speech Therapy and Occupational Therapy, Medical School of the University of São Paulo, São Paulo, Brazil; 3 Neuromuscular Division, Associação de Assistência à Criança Deficiente (AACD), São Paulo, Brazil

**Keywords:** Duchenne muscular dystrophy, Steroids, Vital forced capacity, Respiratory function tests, Myopathies

## Abstract

**Background:**

Duchenne muscular dystrophy (DMD) is a sex-linked inherited muscle disease characterized by a progressive loss in muscle strength and respiratory muscle involvement. After 12 years of age, lung function declines at a rate of 6 % to 10.7 % per year in patients with DMD. Steroid therapy has been proposed to delay the loss of motor function and also the respiratory involvement.

**Method:**

In 21 patients with DMD aged between seven and 16 years, the forced vital capacity (FVC) and the forced expiratory volume in one second (FEV_1_) were evaluated at three different times during a period of two years.

**Results:**

We observed in this period of evaluation the maintenance of the FVC and the FEV_1_ in this group of patients independently of chronological age, age at onset of steroid therapy, and walking capacity.

**Conclusion:**

The steroid therapy has the potential to stabilize or delay the loss of lung function in DMD patients even if they are non-ambulant or older than 10 years, and in those in whom the medication was started after 7 years of age.

## Background

Duchenne muscular dystrophy (DMD) is one of the most common inherited pediatric neuromuscular disorders, affecting 1 in 3,500 live male births. It is an X-linked disorder caused by mutations in the dystrophin gene and is characterized by a progressive decrease in muscle strength and motor function [1,2]. Besides the impairment of motor function, one major problem is the progressive involvement of the respiratory muscles which leads to secondary changes such as atelectasis, decreased lung compliance, ineffective cough and occurrence of repeated infections, as well as imbalance in ventilation-perfusion and hypoxia during sleep [3,4].

Patients with DMD develop a restrictive respiratory pattern with reduction of maximal respiratory pressures and forced vital capacity (FVC) that implicates a risk for respiratory failure and death [2,5]. Consequently, measures of lung function are fundamental methods to monitor the outcome of patients [6]. Pulmonary function of patients with DMD increases until the age of 10 to 12 years and then reaches a plateau. This increase reflects the growth of the entire rib cage, including its muscles and ligaments [7,8]. Following the plateau phase, lung function declines at a rate of 6-8 % annually [6-10]. A recent study shows a decrease of vital capacity of 10.7 % per year in patients with DMD [11].

Steroid therapy has been used to slow the progression of the disease with many studies showing a delay in motor impairment from six months to three years [12-16]. Although it is still uncertain how long steroids can slow the progression of respiratory impairment, retrospective studies of long-term monitoring in patients with DMD have documented the maintenance of lung function for a long period [14,15]. The aim of our study was to evaluate longitudinally the pulmonary function in DMD patients on steroid therapy and its relation to age, motor ability (ambulant or not), and age at the onset of steroid therapy.

## Methods

### Study design

We evaluated 30 boys with DMD who were between seven and 23 years of age. The diagnosis of DMD was confirmed by molecular analysis and/or by imunohistochemistry and Western blot of dystrophin in muscle biopsy. All children were being treated with deflazacort (0.9 mg/kg/day once daily) or prednisolone (0.75 mg/kg per day intermittently 10 days on/10 days off). A control group was not included in this study because most of our patients had submitted to steroid treatment. Patients were excluded from the study in the following circumstances: tracheotomy, smokers, respiratory infection or other pulmonary diseases, and discontinuation of the steroid therapy during the study. The term of consent was signed by the parents and approved by the Ethics Committee for Analysis of Research Projects of our Institution.

Patients were classified according to the age of evaluation [10 years of age or younger (≤10 years), and older than 10 years of age (>10 years)], age of onset of steroid therapy [seven years of age or younger (≤7 years), and older than seven years of age (>7 years)] and walking capacity (ambulant and non-ambulant). The height of ambulant patients was measured in a standing position, with correction of the equines support when possible; when not, height was estimated using the length of the arm span. The tests were performed by the same physiotherapist using a spirometer (microQuark, Cosmed, Italy) coupled to a microcomputer with daily calibration. Three evaluations were performed, all on the days of regular medical appointments. The patients were assessed initially at the first evaluation; and the second and third evaluations, respectively, were performed one and two years later.

### Pulmonary function testing

Measurements were performed with the patient in the sitting position, following the recommendations of the American Thoracic Society and the European Respiratory Society (ATS/ERS) [17]. Up to eight tests were performed, with an interval of rest of one minute or more (if necessary). We selected the three best performances with a maximum variability of 5 % or 200 ml between them. The parameters evaluated were FVC and forced expiratory volume in 1 sec (FEV_1_), both expressed as absolute and relative values. The relative values were obtained by comparison with normal values for all spirometric variables [18]. Statistical analysis was performed using the analysis of variance (ANOVA) with repeated measures to compare the average results obtained in the groups of patients (group effect) and among the three different periods of evaluation (time effect). When the covariance matrix of the observations of the same individual was not found, the tests were adjusted based on Huynh-Feldt correction. An index of significance of 5 % (p < 0.05) was adopted and it was verified if there was interaction among the results.

## Results

Of the 30 patients who were evaluated initially, only 21 were monitored for the entire period of two years. Patients were excluded because they failed to use medication regularly or because they failed to attend the evaluations. The age of the 21 patients that concluded the study varied from 7 to 16 years. The mean age of the first recording was 11.6 ± 3 years. The mean height and weight, respectively, were 142.8 ± 15.0 cm and 35.6 ± 9.1 kg at the first evaluation and 147.8 ± 14.2 cm and 40.7 ± 9.5 kg at the last visit. The mean body mass index was 17.5 ± 3.4 at the first evaluation and 18.7 ± 3.6 at the last visit. The average age at the loss of walking capacity was 10.7 ± 2.7 years. None patient had been submitted to spinal surgery, most of the patients (86 %) attended physiotherapy sections and a small proportion of them (24 %) also attended respiratory therapies. Seven patients (33 %) performed an air stacking maneuver at home and during physical therapy sessions and two (10 %) utilized non-invasive ventilation (NIV) at night. The average age of onset of steroid therapy was 8.05 ± 3.06. Ten patients (48 %) started the medication with ≤7 years and 11 (52 %) after this age. Only two patients started the drug after the loss of walking capacity. One was 16 years old and initially had absolute and relative values of FVC of 0.4 L and 36.9 %, respectively, and at the last visit, 0.89 L and 34.7 %, respectively. The other was 15 years old and initially showed absolute and relative values of FVC of 1.33 L and 43.6 %, respectively, and 1,43 L and 54.9 %, respectively, at the end of follow up.

**Figure 1:**
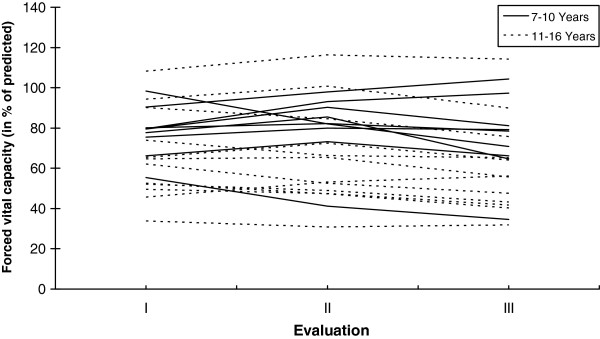
**Individual values of Forced Vital Capacity (FVC) were expressed in % of the predicted value.** After the Evaluation I (baseline), patients were followed yearly (evaluations II and III). Patients were divided in 2 groups according their age (7–10 years and 11–16 years).

**Figure 2:**
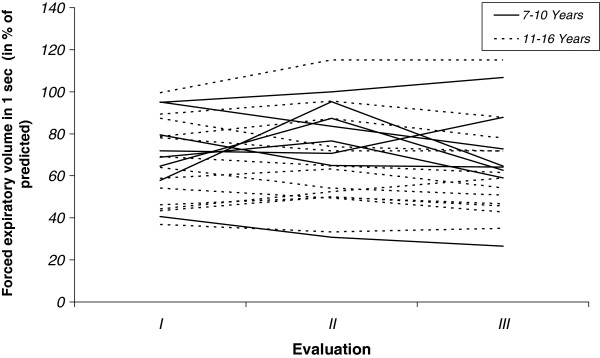
**Individual values of Forced Expiratory Volume in 1 sec (FEV**_**1**_**) were expressed in % of the predicted value.** After the Evaluation I (baseline), patients were followed yearly (evaluations II and III). Patients were divided in 2 groups according their age (7–10 years and 11–16 years).

In relation to the three times of evaluation, the FVC and FEV_1_ expressed in absolute and relative values showed no statistically significant change either in patients ≤10 years or those older than 11 years (Tables 1 and 2, Figure 1 and 2). Also, in patients who started the steroid therapy with ≤7 years and after of this age, as well as in ambulant and non-ambulant patients, no significant changes were observed (Tables 1 and 2). Comparing the groups of patients according to age (≤10 years and >10 years), age of onset of therapy (≤7 years and >7 years), and walking capacity (ambulant and non-ambulant), lung function did not show any statistically significant differences (p > 0.05). In addition, interaction in the results of each group tested was not observed (Tables 1 and 2).

**Table 1:** Forced Vital Capacity (FVC) in three times of evaluation according to the age (≤10 years and >10 years), age of onset of steroid therapy (≤7 years and >7 years), and walking capacity (ambulant and non-ambulant)

		**FVC (L/%)**		
	**Evaluation I**	**Evaluation II**	**Evaluation III**	
Age (≤10 years)	1.6 ± 0.3/	1.7 ± 0.4/	1.7 ± 0.4/	p^1^ = 0.24/0.59
*n* = 9	78.8 ± 12.5	80.1 ± 16.6	75.2 ± 20.2	p^2^ = 0.07/0.31
Age (>10 years)	1.7 ± 0.4/	1.8 ± 0.4/	1.7 ± 0.4/	p^3^ = 0.19/0.09
*n* = 12	66 ± 22.1	65.5 ± 24.7	60.5 ± 23.6	
Age of onset of steroid therapy (≤7 years)	1.6 ± 0.3 / 75.9 ± 16.3	1.7 ± 0.5 / 78.5 ± 22.5	1.7 ± 0.4 / 74.7 ± 25.5	p^1^ = 0.24/0.61
				p^2^ = 0.05/0.25
				p^3^ = 0.21/0.23
*n* = 10				
Age of onset of steroid	1.7 ± 0.4 / 66.9 ± 21.3	1.8 ± 0.4 / 66.1 ± 21.7	1.7 ± 0.3 / 59.6 ± 18.5	
therapy (>7 years)				
*n* = 11				
Ambulant	1.7 ± 0.4 / 86.2 ± 10.6	1.9 ± 0.4 / 92.3 ± 11.5	1.9 ± 0.3 / 87.2 ± 15.6	p^1^ = 0.37/0.65
				p^2^ = 0.41/0.99
*n* = 9				p^3^ = 0.83/0.67
Non-ambulant	1.6 ± 0.4 / 60 ± 16.3	1.6 ± 0.4 /56.7 ± 15.1	1.5 ± 0.3 / 51.5 ± 13.3	
*n* = 12				

Table legend: p^1^ = comparison between the three evaluations, p^2^ = comparison between both groups, p^3^ = interaction effect.

**Table 2:** **Forced Expiratory Volume in 1 sec (FEV**_**1**_**) in three times of evaluation according to the age (≤10 years and >10 years), age of onset of steroid therapy (≤7 years and >7 years), and walking capacity (ambulant and non-ambulant)**

		**FEV**_**1 **_**(L/%)**		
	**Evaluation I**	**Evaluation II**	**Evaluation III**	
Age (≤10 years)	1.3 ± 0.3/72.5 ± 17.14	1.5 ± 0.5/77.4 ± 20.8	1.3 ± 0.4/69.1 ± 22	p^1^ = 0.35/0.43
*n* = 9				p^2^ = 0.16/0.17
Age (>10 years)	1.5 ± 0.4/64.3 ± 20.6	1.5 ± 0.3/64.5 ± 22.5	1.5 ± 0.3/61.8 ± 22.3	p^3^ = 0.13/0.16
*n* = 12				
Age of onset of steroid	1.3 ± 0.3 / 71.6 ± 19.1	1.5 ± 0.5 / 76.2 ± 24.8	1.4 ± 0.5 / 72.1 ± 26.6	p^1^ = 0.32/0.38
therapy (≤7 years)				p^2^ = 0.05/0.22
				p^3^ = 0.18/0.18
*n* = 10				
Age of onset of steroid	1.5 ± 0.4 / 64.3 ± 19.6	1.5 ± 0.3 / 64.3 ± 19	1.4 ± 0.2 / 58.5 ± 15.2	
therapy (>7 years)				
*n* = 11				
Ambulant	1.4 ± 0.3 / 80.4 ± 13.9	1.6 ± 0.4 / 87.8 ± 15.9	1.6 ± 0.3 / 82.1 ± 19.1	p^1^ = 0.42/0.41
				p^2^ = 0.18/0.98
*n* = 9				p^3^ = 0.50/0.38
Non-ambulant	1.4 ± 0.4 / 58.3 ± 17.5	1.4 ± 0.4 / 56.7 ± 16.1	1.3 ± 0.3 / 52.2 ± 13.9	
*n* = 12				

Table legend: p^1^ = comparison between the three evaluations, p^2^ = comparison between both groups, p^3^ = interaction effect.

## Discussion

Our study demonstrated the maintenance of lung function for a period of up to two years in patients with DMD treated with steroids regardless of age, walking capacity, and age of onset of therapy. We were not able to confirm that steroid therapy alone is responsible for the maintenance of lung function, due to the lack of a control group. According to McDonald et al., [9] the loss of walking ability and spinal deformities can affect lung function but age is the most important factor. Regarding age, we have detected that in patients older than 10 years, lung function was maintained without significant changes during a period of up to two years, while in the natural history of disease, lung volumes typically decline about 6 % to 10.7 % per year after 10 years of age [6,9-11]. This finding clearly demonstrated the efficacy of the steroid therapy in delaying respiratory deterioration in these patients, even when they are older than 10 years of age.

Steroids conventionally are used in order to increase the walking period. However, some authors have suggested that the treatment should be continued in wheelchair patients due to the potential effect of the medication in preventing spinal deformity and the deterioration of respiratory function [15,19-21]. In our study, both wheelchair-bound patients and ambulant patients showed stability of the respiratory parameters, which demonstrates the maintenance of lung function in steroid-treated patients, even after the loss of walking capacity.

The optimal age to begin steroid treatment in order to preserve respiratory function is poorly defined. In theory, the use of the steroids at the initial stages of the disease could prevent the development of disabilities, including the deterioration of lung function. We evaluated respiratory function in patients who started steroid therapy ≤7 years and >7 years of age in order to verify the possible influence of the age of onset of therapy on the evolution of lung function. Both groups showed stability of FVC and FEV_1_, which indicates that steroid treatment might stabilize lung function even in those children who are first treated when they are older than 7 years of age. Longer follow-up is necessary to clarify the best time to start the medication.

Although not as sensitive as the maximum inspiratory pressure (MIP) and maximum expiratory pressure (MEP), FVC and FEV_1_ give good information on the reduction of pulmonary compliance and chest wall dynamic, which reflects the state of the bronchi and requiring muscle power to obtain maximum values. Therefore, FVC and FEV_1_ provide useful and reproducible measures of the progression of the disease [3,6]. Furthermore, FEV_1_ and MIP are strongly correlated [10,11]. Possibly, a longer follow-up would determine how long pulmonary function and respiratory muscle strength remain stable in DMD patients treated with steroids. Some of our patients performed the “air stacking” maneuver at home or during physiotherapy. However, we did not determine how frequently the maneuver was performed or the influence of the maneuver on pulmonary parameters. The “air stacking” maneuver is reported to have positive effects only in peak flow of cough and maximum insufflation capacity [22,23]. The CVF continued to decline independently of the use of this maneuver [23]. Only a small number of our patients used non-invasive ventilation (NIV) during sleep. Therefore, we could not analyze the effects of NIV.

This study present 2 limitations: first, the period of follow up may be considered short (2 years); however, most study evaluating the effect of corticosteroid in motor impairment in DMD patients have studied such period of time [20,21]. Second, the absence of control could represent a limitation; however, there are studies demonstrating that corticosteroid improves motor function in DMD patients and for ethical reasons a control group was considered inadequate by our hospital´s ethical committee.

## Conclusion

Our study has shown that steroid treatment has the potential to stabilize lung function in DMD patients even in non-ambulant children, in those older than 10 years and in those in which the medication was started after 7 years of age.

## Abbreviations

DMD: Duchenne muscular dystrophy; FVC: Forced vital capacity; FEV1: Forced expiratory volume in one second; MIP: Maximum inspiratory pressure; MEP: Maximum expiratory pressure; NIV: Non-invasive ventilation.

## Authors’ contribution

DLM: study design and conception; data acquisition, analysis and interpretation; manuscript elaboration. ECS: data acquisition. MBDR: substantial contribution to study conception and data acquisition. CRFC: substantial contribution to study conception; data acquisition, analysis and interpretation. EZ and UCR: substantial contribution to study conception and data acquisition; manuscript elaboration; critical revision and approval of the manuscript final version. All authors read and approved the final manuscript.

## Competing interests

The authors declare that they have no competing interests.
